# Macro- and Micro-Level Behavioral Patterns in Simulation-Based Scientific Inquiry: Linking Processes to Performance Among Elementary Students

**DOI:** 10.3390/jintelligence14010006

**Published:** 2026-01-04

**Authors:** Shuang Wang, An Hu, Lu Yuan, Wei Tian, Tao Xin

**Affiliations:** 1 Collaborative Innovation Center of Assessment for Basic Education Quality, Beijing Normal University, Beijing 100875, China; wangshuang_psy@mail.bnu.edu.cn; 2 State Key Laboratory for Artificial Microstructure and Mesoscopic Physics, School of Physics, Peking University, Beijing 100871, China; huan17@pku.edu.cn; 3 Beijing Academy of Educational Sciences, Beijing 100036, China; yuanlupsy@163.com

**Keywords:** scientific inquiry, simulation-based assessment, process data, sequential pattern mining, process mining

## Abstract

Scientific inquiry is fundamental to science education, encompassing the processes through which students construct scientific knowledge and develop thinking skills. However, the unfolding of these inquiry processes and their relation to performance remain underexplored. Drawing on process data from a structured simulation-based assessment task, this study investigated the inquiry processes of 259 fourth-grade students. We applied a multi-analytic approach including sequential pattern mining, entropy analysis, and process mining to capture macro- and micro-level behavioral patterns and examine their associations with task performance operationalized by effectiveness and efficiency. Macro-level analyses revealed that effective students generally organized their inquiry processes into more iterative cycles of evidence collection, demonstrating a more dedicated approach before committing to a final response. Micro-level analyses further indicated that effective and efficient students showed better strategic coordination during experimentation. Together, these findings provide a multi-level characterization of elementary students’ scientific inquiry processes and link inquiry patterns to task effectiveness and efficiency. The study also underscores the potential of process data from simulation-based assessments for diagnosing inquiry skills and informing the design of personalized scaffolds in elementary science education.

## 1. Introduction

Scientific inquiry serves as a cornerstone of science education, equipping students with the ability to generate and evaluate evidence to address scientific problems (Rönnebeck et al., 2016). Engagement in such inquiry can support students’ understanding of core scientific concepts (National Research Council, 2012), foster the development of science process skills (Ekici & Erdem, 2020), and cultivate scientific reasoning and thinking skills (Kant et al., 2017; Lazonder & Kamp, 2012). Beyond the production of final products, scientific inquiry comprises dynamic cycles of generating ideas, collecting and evaluating evidence, and refining ideas (Reith & Nehring, 2020; Rönnebeck et al., 2016). Elucidating these inquiry processes is essential for advancing science education (Osborne & Allchin, 2025) and informing the design of next-generation science assessments (Vo & Simmie, 2025).

Traditional assessments of scientific inquiry have long been dominated by paper-and-pencil tests. These approaches primarily emphasize inquiry products, such as response correctness or total scores, rather than the inquiry processes that lead to them. Although hands-on laboratory activities can support formative assessment of inquiry processes, they are resource-intensive and face practical challenges in large-scale implementation, data collection, and valid scoring (Garcia-Mila et al., 2011; Kruit et al., 2018). Consequently, they often provide limited insight into the temporal unfolding of inquiry. Over the past few decades, simulation-based tasks have emerged as viable alternatives for assessing and developing scientific inquiry skills (S. Chen, 2010; DeBoer et al., 2014; Schellinger et al., 2017). These tasks situate students in interactive and authentic inquiry environments while unobtrusively logging their behaviors as process data (Anghel et al., 2024; Quellmalz et al., 2012), offering rich information about how inquiry unfolds over time (Greiff et al., 2015).

Despite growing interest in using process data from simulation-based inquiry tasks to investigate students’ scientific inquiry processes, several important research gaps remain. First, many existing studies reduce rich process logs into relatively static, aggregate metrics (Gobert et al., 2015; Teig et al., 2020; Wang et al., 2023), such as counts of specific behaviors (e.g., number of trials) and overall time-based metrics (e.g., total time on task). Because these metrics collapse information across behavior sequences, they may not sufficiently capture the dynamic and temporal structure of inquiry processes. Second, while a growing body of research has moved beyond aggregate metrics to examine behavior sequences, studies tend to examine these in isolation. Some studies focus on macro-level patterns describing broad inquiry phases, such as hypothesis generation and experimentation (C. M. Chen & Wang, 2020), whereas others capture micro-level patterns of fine-grained behaviors within these inquiry phases (Teig, 2024). However, relatively few studies have integrated these two levels of granularity to provide a comprehensive account of inquiry processes. Third, although many studies link inquiry processes to task performance, most focus on effectiveness, typically operationalized as task correctness or related scores (R. S. J. D. Baker & Clarke-Midura, 2013; C. M. Chen & Wang, 2020), whereas fewer examine efficiency. When efficiency is considered, it is often indexed by metrics such as task completion time or minimization of redundant behaviors (Gong et al., 2023; Taub et al., 2018). Jointly considering effectiveness and efficiency may help identify specific constellations of inquiry behaviors that characterize productive inquiry.

Therefore, this study investigates how elementary students’ scientific inquiry processes in a simulation-based inquiry task relate to task performance. Specifically, we analyze macro- and micro-level inquiry patterns and treat effectiveness and efficiency as complementary performance dimensions, elucidating not only whether students reach correct solutions but also how they organize and execute their inquiry.

## 2. Related Work

### 2.1. Scientific Inquiry Processes and Cognitive Strategy Use

Scientific inquiry is characterized by dynamic processes encompassing hypothesis formation, evidence collection, evidence evaluation, and conclusion drawing (Emden & Sumfleth, 2016; Pedaste et al., 2015). Hypothesis formation entails proposing tentative explanations or predictions for scientific problems that are grounded in prior knowledge and existing theoretical frameworks. Evidence collection refers to designing and conducting experiments to gather empirical data. Evidence evaluation requires critically examining the extent to which the data support or refute the hypotheses, demanding rigorous thinking to manage uncertainties. Conclusion drawing then synthesizes the available evidence to reach sound inferences.

These phases rarely unfold in a fixed sequence; instead, inquiry processes are inherently dynamic and cyclic as individuals test, revise, and refine their ideas through repeated cycles of experimentation (Metz, 2004; Pedaste et al., 2015). In educational settings, such as laboratory activities, students often navigate recursively between these inquiry phases, revising hypotheses considering new evidence, refining experimental designs to rule out alternative explanations, and updating conclusions as they gather additional evidence (Rönnebeck et al., 2016). The quality of inquiry therefore depends not only on whether students can perform each phase in isolation, but also on how they orchestrate these phases over time (Kranz et al., 2023). These transitions among inquiry phases can provide an overarching macro-level characterization of inquiry processes (Emden & Sumfleth, 2016).

At a fine-grained micro-level, students’ inquiry performance also depends on the strategies they use within and across inquiry phases. Scientific inquiry strategies are cognitive approaches that guide how individuals generate, test, and evaluate ideas considering empirical evidence (Zimmerman, 2007). A well-known example is the control-of-variables strategy (CVS), which supports informative experimentation by strategically varying one variable at a time while holding others constant to establish causal relations (Z. Chen & Klahr, 1999). In multivariate contexts, more flexible coordination among several variables is required (Kuhn et al., 2008), such as systematically exploring combinations of factors or focusing on theoretically relevant contrasts. Prior research indicates that such systematic, evidence-oriented strategies tend to facilitate the generation of conclusive evidence and warranted conclusions, whereas unsystematic strategies such as ill-structured trial-and-error often produce uninformative evidence (Z. Chen & Klahr, 1999; Kuhn et al., 2008). Moreover, differences in students’ inquiry strategies often lead to divergent inquiry products, even when students exhibit similar levels of overt engagement in inquiry environments (e.g., Taub et al., 2018; Teig, 2024; Ulitzsch et al., 2022).

### 2.2. Scientific Inquiry Processes and Performance in Simulation-Based Inquiry Tasks

Simulation-based inquiry tasks provide interactive, computer-based environments where students can design and conduct experiments while their behaviors are unobtrusively logged as process data (Bergner & von Davier, 2019; DeBoer et al., 2014; Quellmalz et al., 2012). These process data capture fine-grained inquiry behaviors, such as adjusting variables and running trials, enabling researchers to reconstruct students’ behavioral trajectories and analyze the underlying inquiry processes (H. Li et al., 2018; Wang et al., 2023).

Process data can be analyzed at multiple levels of granularity, facilitating the investigation of both overarching and fine-grained characteristics of students’ inquiry processes (Goldhammer et al., 2021; Lindner & Greiff, 2023). At the macro level, inquiry processes are typically represented as state sequences of inquiry phases (Emden & Sumfleth, 2016; Reith & Nehring, 2020), such as hypothesis generation, evidence collection, evidence evaluation and conclusion drawing, which delineate the overall progression of students’ inquiry (C. M. Chen & Wang, 2020). These representations reveal, for instance, whether students engage in iterative cycles of experimentation and evaluation or exhibit premature closure by jumping to conclusions. At the micro level, inquiry processes are represented as event sequences of discrete behaviors, for example, running trials and revising answers. Analyses at this level enable the characterization of how students organize their behaviors into inquiry strategies within and across inquiry phases (Stadler et al., 2024; Teig, 2024; Xu et al., 2024). Micro-level analyses are particularly valuable for detecting within-phase dynamics and short-range behavioral dependencies that remain obscured by macro-level phase summaries.

To contextualize inquiry processes meaningfully, researchers have increasingly linked behavioral patterns with task performance (Chiou et al., 2022; van Dijk et al., 2016; Wen et al., 2020; J. Zheng et al., 2019). Studies comparing students with different levels of inquiry effectiveness (typically based on whether students reach valid conclusions) show that effective students engage in more systematic and coherent experimentation cycles. For example, using process data from large-scale computer-based inquiry tasks, Teig (2024) found that high-performing students were able to apply the control-of-variables strategy effectively in univariable contexts and flexibly coordinate several variables in multivariable tasks, whereas low-performing students often manipulated several variables at once, repeated similar trials, or executed random behaviors without changing variables. Similarly, using process data from a buoyancy simulation in the web-based inquiry environment, C. M. Chen and Wang (2020) identified behavioral patterns differentiating students with varying levels of inquiry performance, showing that high-performing students frequently engaged in cycles of running the experiments and renewing their hypotheses, whereas low-performing students seldom engaged in such cycles.

Beyond effectiveness, efficiency reflects the economy with which students utilize their time and cognitive effort to reach a conclusion. Taub et al. (2018) analyzed process data from Crystal Island, a game-based environment for microbiology, and operationalized efficiency via the number of diagnosis worksheet submissions. They found that efficient students solved the problem in fewer attempts, tested fewer partially relevant and irrelevant items, and engaged in fewer testing sequences than less efficient students. Together, these studies demonstrate that in simulation- and game-based inquiry tasks, inquiry performance can be conceptualized as a multidimensional construct encompassing both effectiveness (i.e., task correctness) and efficiency (e.g., the number of steps, attempts, or time required to reach a conclusion).

### 2.3. The Present Study and Research Questions

Building on prior research, this study investigates elementary students’ scientific inquiry processes in a simulation-based assessment environment. We examine how inquiry patterns at both macro and micro levels relate to task performance, which is operationalized along two dimensions: effectiveness (task correctness) and efficiency (task completion time). Specifically, this study aims to address the following research questions:

RQ1: At the macro level, how do effective and less effective students differ in their overall inquiry processes?

RQ2: At the micro level, within effectiveness groups, how do efficient and less efficient students differ in their inquiry processes?

**Figure 1:**
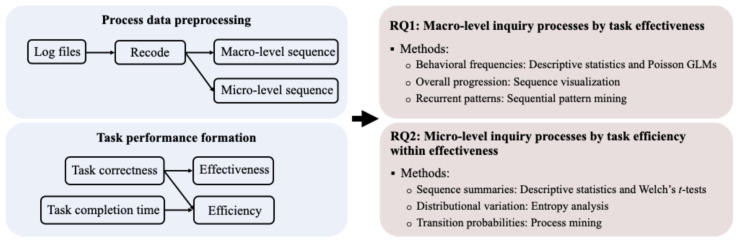
The analytical framework of this study.

The analytical framework of this study is illustrated in Figure 1. Log files were preprocessed and recoded into macro- and micro-level behavior sequences. Task performance was operationalized along two dimensions and used to form performance subgroups. To derive a comprehensive, multi-level characterization of students’ inquiry processes, we employed a multi-analytic approach: (1) Macro-level inquiry processes (RQ1) were analyzed using descriptive statistics, Poisson generalized linear models (GLMs), sequence visualization, and sequential pattern mining; (2) Micro-level inquiry processes (RQ2) were examined using descriptive statistics, entropy analysis, and process mining.

## 3. Methods

### 3.1. Participants and Procedure

**Figure 2:**
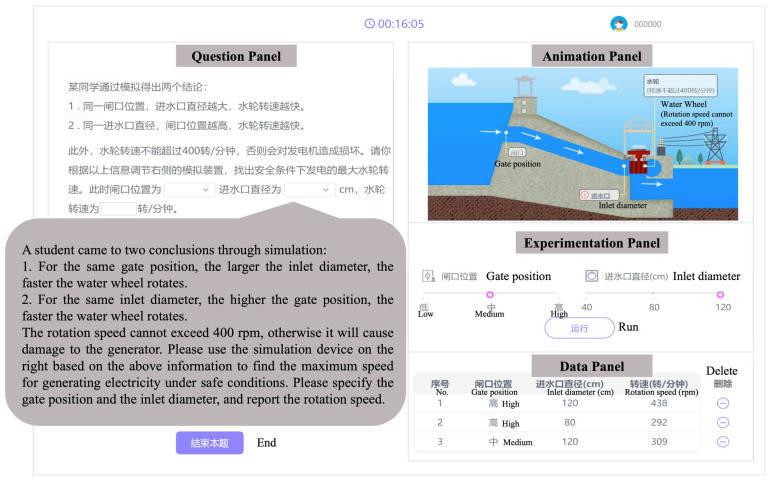
Illustrative user interface for the Hydroelectric Power Plant task. This schematic mock-up was adapted from the original interface for publication and copyright reasons; the layout and functions are equivalent to those in the version used in the study.

The analytical sample comprised 259 fourth-grade students (*M_age_* = 10.21 years, *SD* = 0.67, range = 8–13 years) from four public elementary schools in southern China. These students participated in a structured, computer-based assessment conducted in 2019 as part of a large-scale educational project on scientific inquiry skills administered by the Collaborative Innovation Center of Assessment for Basic Education Quality at Beijing Normal University. As part of the assessment, students completed a simulation-based inquiry task titled Hydroelectric Power Plant (see Figure 2). All students completed the task, with approximately 33% successfully arriving at the correct solution. None of the students had prior experience with simulation-based scientific inquiry tasks. Prior to the formal assessment, students received an introduction to the task environment and interface and were allotted up to 10 min of hands-on familiarization to ensure proficiency with the interactive functions. The present study utilized fully de-identified log files from this task, and no additional data collection or direct contact with the students or schools was involved.

### 3.2. Simulation-Based Inquiry Task: Hydroelectric Power Plant

The Hydroelectric Power Plant task focuses on scientific experimentation, requiring students to design and conduct experiments and to evaluate experimental evidence and solve a science problem. In this task, students were asked to determine the optimal operating condition for an electric generator that yields the maximum possible rotation speed while maintaining the speed below a safety limit of 400 revolutions per minute (rpm). The simulation involves manipulating two independent variables: gate position (Low, Medium, and High), and inlet diameter (40 cm, 80 cm, and 120 cm). The default setting initializes with the gate position is Low and inlet diameter is at 40 cm.

Figure 2 presents the user interface of the Hydroelectric Power Plant task, which includes four main panels: the Question Panel (left) poses the problem statement and reiterates two qualitative relationships (priors) between variables and rotation speed; the Animation Panel (upper right) provides a visual representation of the hydropower system; the Experimentation Panel (middle right) allows students to manipulate variables and run trials; and the Data Panel (lower right) automatically records the rotation speed returned by the simulation for each trial.

To solve this multivariate problem, students designed and conducted experiments by adjusting the variable settings and observing the experimental record. They evaluated the collected evidence to identify the optimal experimental condition. They were required to submit a single answer specifying the gate position, the inlet diameter, and the corresponding rotation speed. If any of the three elements were left blank, the system prompted students to complete all elements before submitting their final answer.

**Figure 3:**
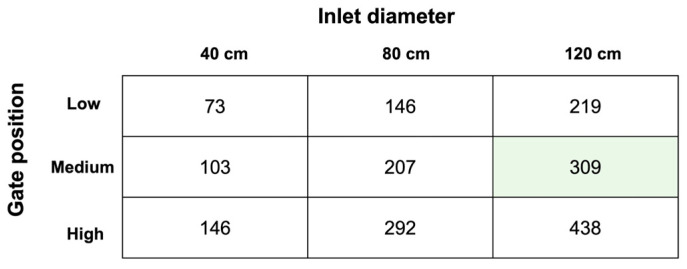
Water wheel rotation speed (rpm) for each combination of gate position and inlet diameter. The cell with a green background indicates the unique optimal solution (i.e., the correct answer).

Figure 3 displays the rotation speed (rpm) for each combination of inlet diameter and gate position. Task correctness was defined as identifying the specific combination that maximizes rotation speed subject to the safety constraint (<400 rpm). Under this constraint, the setting with a 120 cm inlet diameter and a Medium gate position (309 rpm) represents the unique optimal solution. Cognitively, identifying this condition required comparing at least three candidate conditions: the optimal setting (Medium/120 cm, 309 rpm), the setting exceeding the limit (High/120 cm, 438 rpm), and the next best safe setting (High/80 cm, 292 rpm). Task correctness was scored dichotomously: coded as 1 only if all three submitted elements were correct, and 0 otherwise.

### 3.3. Behavior Coding Scheme

To capture students’ inquiry behaviors and ensure the interpretability of process data, we developed a two-level behavior coding scheme. The scheme was grounded in established theoretical frameworks of scientific inquiry (Emden & Sumfleth, 2016; Pedaste et al., 2015) and aligned with the structured interface of the Hydroelectric Power Plant task.

*Micro-Level Coding*. All process logs were first extracted as interface-level events capturing the operational flow. In the Experimentation Panel, the log recorded adjustments to gate position, adjustments to inlet diameter, and clicks on the “Run” button. In the Data Panel, clicks on the “Delete” button that removed an existing data row were recorded. In the Question Panel, response-related events were edited to comprise three answer elements (two dropdown lists and one text box). The system also logged when students entered the task and when they clicked the “End” button to submit their final responses.

Based on these interface-level events, two coders with graduate-level training in psychological measurement developed a rule-based coding scheme for micro-level behaviors. Because the focal question contained three elements, answer events were defined at the item level rather than for individual answers. For each student, all edits to any of the three elements were grouped into answer episodes. The first episode in which the student provided any response was coded as Initial Answer. Any later episode in which the student returned to the item and modified at least one element was coded as Revise Answer. Within an episode, all edits were collapsed into a single event labeled as Initial Answer or Revise Answer so that the code reflected meaningful item-level attempts and revisions.

*Macro-Level Aggregation and Reliability.* These micro-level codes were subsequently aggregated into the macro-level behaviors. The structured layout of the task interface allowed these behaviors to be mapped onto three cognitive components: Evidence Collection (DESIGN and CONDUCT), Evidence Evaluation (ANSWER and MANAGE), and Task Control (START and END).

To establish the coding scheme, the coders resolved initial ambiguities through discussion until full consensus on the coding rules was reached. Following this, the coding was automated using Python scripts. To assess inter-rater reliability, the two coders manually coded a random sample of 50 students (625 log events) into both macro-level and micro-level categories using the final coding scheme (Table 1), yielding near-perfect agreement (Macro-level: Cohen’s κ = 1.00, 100% agreement; Micro-level: Cohen’s κ = 0.98, 98.6% agreement).

Table 1 details the final behavior coding scheme. For instance, within Evidence Collection, DESIGN behaviors are captured by micro-level codes such as Adjust Gate and Adjust Diameter, while CONDUCT behaviors include running trials (Run Trial) with specific Gate × Diameter conditions automatically recorded in the Data Panel. Evidence Evaluation codes capture students’ behaviors related to initial response (Initial Answer), revising responses (Revise Answer) and managing data (Remove Record). Task Control marks the initiation (Start Task) and completion (End Task) of the task.

### 3.4. Statistical Analyses

We employed a multi-analytic approach that was organized into four stages to address the research questions. First, raw log files were preprocessed to construct macro- and micro-level behavior sequences. Second, performance subgroups were established by stratifying students based on task effectiveness (task correctness) and efficiency (task completion time). Third, to address RQ1, we examined macro-level inquiry patterns using descriptive statistics, Poisson GLMs, sequence visualization, and sequential pattern mining. Finally, to address RQ2, we analyzed micro-level inquiry dynamics using entropy analysis and process mining. The specific procedures and rationale for each stage are detailed below.

#### 3.4.1. Process Data Preprocessing

**Figure A1:**
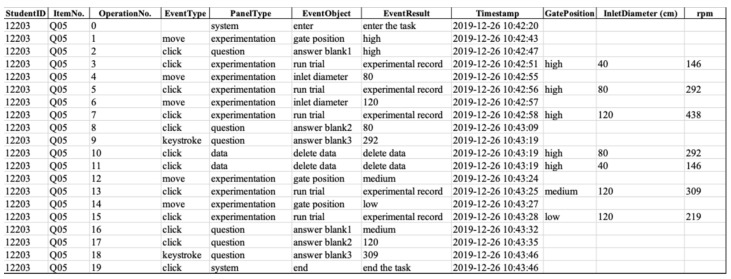
Example of a student’s log file for the Hydroelectric Power Plant task.

Initially, raw log files were chronologically ordered and recoded into macro- and micro-level behavior categories, adhering to the coding scheme in Table 1. For each student, this process yielded two parallel time-ordered sequences: a macro-level sequence representing the progression of broad inquiry phases, and a micro-level sequence capturing fine-grained interface interactions. These recoded sequences served as the basis for all subsequent analyses (see Figure A1 for an example of one student’s log file).

#### 3.4.2. Task Performance Subgroup Formation

**Figure 4:**
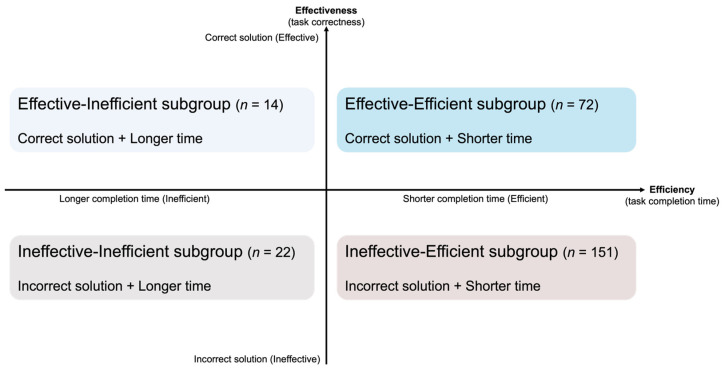
Performance subgroups formation based on effectiveness (task correctness) and efficiency (task completion time).

Subsequently, the derived behavior sequences were stratified based on students’ task performance, which was operationalized along two dimensions: effectiveness (task correctness) and efficiency (task completion time). Figure 4 provides a schematic overview of the four resulting performance subgroups.

*Effectiveness Classification.* Students were first classified into two effectiveness groups based on their final solutions: the Effective group (*n* = 86), comprising students who successfully identified the unique optimal condition, and the Ineffective group (*n* = 173), comprising those who did not.

*Efficiency Classification*. In order to identify efficiency profiles, we applied Gaussian mixture models (GMMs) to task completion time (in seconds) within each effectiveness group. We fitted models with *K* = 1, 2, 3, and 4 components and evaluated model fit using the Bayesian Information Criterion (BIC) and Akaike Information Criterion (AIC) (see Table A1). For the Effective group, the two-component solution (*K* = 2) yielded the lowest BIC and AIC values. For the Ineffective group, the two-component solution minimized BIC, whereas AIC slightly favored *K* = 3. We selected the two-component solution for both groups because it minimized BIC, produced a clear separation between Efficient (faster) and Inefficient (slower) students with high classification certainty (Mean maximum posterior probability: Effective = 0.95; Ineffective = 0.91), and produced consistent and interpretable efficiency profiles across effectiveness groups.

Within the Effective group, the two components corresponded to an Effective–Efficient subgroup (*M* = 81.00, *SD* = 28.16; *n* = 72, 84% of students) and an Effective–Inefficient subgroup (*M* = 183.19, *SD* = 54.54; *n* = 14, 16% of students). Within the Ineffective group, the two components corresponded to an Ineffective–Efficient subgroup (*M* = 61.73, *SD* = 20.24; *n* = 151, 87% of students) and an Ineffective–Inefficient subgroup (*M* = 117.12, *SD* = 50.90; *n* = 22, 13% of students).

Furthermore, to validate the robustness of these efficiency profiles, we conducted a sensitivity analysis using alternative grouping methods (median splits and tertiles), and a behavioral proxy for efficiency—the proportion of Adjust Diameter to Run Trial transitions ($P_{DR}$). As shown in Table A2, the Inefficient profiles consistently exhibited higher $P_{DR}$ than the Efficient profiles. Furthermore, in a regression of $P_{DR}$ on standardized log completion time and task correctness, longer completion time significantly predicted higher $P_{DR}$ (b = 0.03, *p* < 0.001, R² = 0.13). Together, the consistency across grouping methods and the association with the behavioral proxy support the robustness of the GMM-based efficiency profiles.

#### 3.4.3. Macro-Level Analyses

Focusing on macro-level inquiry patterns (RQ1), we employed three analytical techniques: descriptive statistics and Poisson GLMs, sequence visualization, and sequential pattern mining.

*Descriptive Statistics and Poisson GLMs*. We first summarized macro-level behavioral frequencies using groupwise means and standard deviations. To compare across groups, we fitted separate Poisson GLMs with a log link for each macro-level behavior, using effectiveness group (Effective vs. Ineffective) as the predictor and the log of total macro-level behaviors (sequence length) as an offset. This specification models behavior counts as rates per step, thereby controlling for individual differences in total sequence length. To improve inference robustness against potential heteroskedasticity and mild overdispersion, we used HC3 heteroskedasticity-robust standard errors. We report Incidence Rate Ratios (IRRs) with 95% Wald confidence intervals, computed by exponentiating the estimated coefficients and their HC3-based confidence limits. To account for multiple comparisons across behaviors, all *p*-values were adjusted using the Benjamini–Hochberg false discovery rate (FDR) procedure (Benjamini & Hochberg, 1995).

*Sequence Visualization*. We generated sequence distribution plots for each effectiveness group, aiming to provide a qualitative overview of macro-level inquiry progression. These visualizations depict the cross-sectional distribution of macro-level states at each time step, illustrating group-level trends across the entire task timeline.

*Sequential Pattern Mining*. The cSPADE algorithm (Zaki, 2001) was utilized to identify recurrent macro-level behavioral subsequences, which extracts sequential patterns occurring above a predefined support threshold. We first ran cSPADE separately for the Effective and Ineffective groups and defined the candidate pattern set as the union of subsequences with support ≥ 0.30 in at least one group. For each candidate subsequence, we treated each student as a Bernoulli trial (pattern present vs. absent) and computed 95% confidence intervals for group-specific support using Wilson score intervals for binomial proportions. To compare differences across effectiveness groups, we conducted two-proportion z tests and adjusted the resulting *p* values using the Benjamini–Hochberg procedure (Benjamini & Hochberg, 1995). In this study, we retained only subsequences with support ≥ 0.30 and FDR-adjusted *p* < 0.05, and excluded single-event subsequences. The resulting patterns can reflect typical sequential characteristics of students’ macro-level inquiry processes.

#### 3.4.4. Micro-Level Analyses

Focusing on micro-level patterns (RQ2), the fourth stage examined behavioral patterns at the micro level, complementing the macro-level analyses by focusing on the diversity and order of fine-grained behaviors within the task. Three analytical techniques were applied: descriptive statistics, entropy analysis and process mining.

*Descriptive Statistics.* We first characterized micro-level sequences using summary metrics, including task completion time and average sequence length. Group differences between Efficient and Inefficient profiles within each effectiveness group were examined using Welch’s *t*-tests to account for unequal variances.

*Entropy Analysis.* Shannon entropy was calculated to quantify the diversity of behavioral repertoires. For each student, the relative frequencies of distinct micro-level behaviors (e.g., Adjust Gate, Run Trial) were treated as probabilities $p_{i}$, and the entropy value (*H*) was computed as:

(1) $$H=-\sum_{i}p_{i}{log}_{2}p_{i}$$

Higher entropy values indicate more diverse and exploratory set of behaviors, whereas lower values suggest repetitive or narrowly focused behaviors. To ensure comparability across students, we computed normalized entropy by dividing *H* by the maximum possible entropy (${log}_{2}K$, where K = the number of distinct behaviors, here K = 8), yielding an index ranging from 0 to 1. For each performance subgroup, we summarized normalized entropy using the mean and 95% bootstrap confidence intervals based on 2,000 resamples. Group differences were examined using a two-way ANOVA with effectiveness, efficiency, and their interaction as between-subject factors, and partial eta squared was reported as the effect size. Visual inspection of residuals indicated no severe deviations from normality or homoscedasticity.

*Process Mining*. Following previous work (Tan et al., 2025; Teig, 2024), to characterize the temporal structure and sequential dependencies of micro-level inquiry behaviors, we used process mining to model transitions between behaviors. We employed first-order Markov models based on transition probabilities to identify structured process models that capture frequent and distinctive transitions. Process models represented behaviors as nodes and transitions as directed edges weighted by transition probabilities. For each performance subgroup, all consecutive behavior pairs were aggregated to compute edge frequencies and conditional transition probabilities:

(2) $$P\left(t|s\right)=count(s,t)/\sum_{t^{\prime }}count\left(s,t^{\prime }\right)$$

where s and t denote the source and target behaviors, respectively.

To mitigate estimation bias arising from small subgroup sizes and sparse transitions, we estimated smoothed transition matrices separately for each performance subgroup using a hierarchical Dirichlet–multinomial approach. A global transition matrix from the full sample served as a prior (concentration parameter α = 0.5), which provides a weakly informative prior that mildly shrinks rare transitions toward the overall pattern while preserving frequent, subgroup-specific transitions.

We quantified uncertainty using a non-parametric bootstrap procedure with 1000 resamples with replacement. Transition probabilities are reported with their 95% bootstrap confidence intervals. To visualize the dominant patterns, process models display only edges with transition probabilities *Pr* ≥ 0.30. Finally, to examine efficiency differences conditional on effectiveness, we generated differential transition diagrams (delta probability maps). These maps visualize the difference in transition probabilities between efficient and less efficient students within each effectiveness group. Statistical significance of these differences was determined using 95% bootstrap confidence intervals of the delta values.

#### 3.4.5. Analytical Environment

All statistical analyses and data processing were conducted in Python (version 3.9.23; Van Rossum & Drake, 2009). Data manipulation and preprocessing relied on NumPy (version 2.0.2) and pandas (version 2.3.2), while statistical modeling—including Poisson GLMs and entropy computations—was implemented using SciPy (version 1.13.1) and statsmodels (version 0.14.5). GMMs were fitted using the GaussianMixture class from scikit-learn (version 1.4.2), and sequential pattern mining utilized the pycspade library (version 0.6.6). For sequence visualization, we developed custom Python scripts designed to replicate the sequence distribution plots of the TraMineR package (version 2.2-12) in R (version 4.5.1; R Core Team, 2024). Visualizations were generated using Matplotlib (version 3.9.4).

To ensure computational reproducibility, particularly for procedures involving stochastic components (e.g., GMM initialization and bootstrap resampling), we fixed the global random seed to 0 (np.random.seed(0)) and constrained parallel processing by setting the environment variable OMP_NUM_THREADS = 1.

## 4. Results

### 4.1. Macro-Level Inquiry Processes Across Effectiveness Groups (RQ1)

To address RQ1, we first compared macro-level behavioral frequencies across effectiveness groups using descriptive statistics and Poisson GLMs (Table 2). The results indicated clear group differences. Relative to the Ineffective group, the Effective group showed significantly higher rates of designing experiments (DESIGN: IRR = 1.53, 95% CI [1.38, 1.69], *p* < 0.001) and conducting experiments (CONDUCT: IRR = 1.30, 95% CI [1.19, 1.42], *p* < 0.001). They also engaged more frequently in data management (MANAGE: IRR = 1.88, 95% CI [1.10, 3.20], *p* = 0.020). Conversely, they submitted answers significantly less frequently than the Ineffective group (ANSWER: IRR = 0.56, 95% CI [0.48, 0.65], *p* < 0.001).

**Figure 5:**
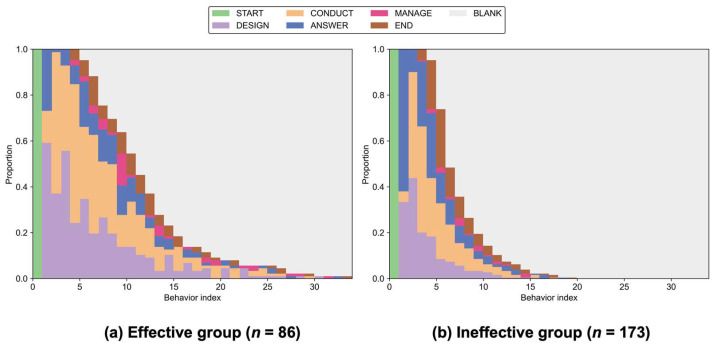
Sequence plots of macro-level inquiry progression for (**a**) the Effective group and (**b**) the Ineffective group. Abbreviations: START = Start the task; DESIGN = Design experiments; CONDUCT = Conduct experiments; ANSWER = Answer the question; MANAGE = Manage data; END = End the task; BLANK = Filler state representing sequence positions shorter than the longest behavior sequence.

Figure 5 illustrates sequence visualizations of macro-level inquiry processes for the two effectiveness groups. In both groups, experimentation behaviors (DESIGN and CONDUCT) dominated the central portions of the sequences, reflecting the fundamental role of experimentation in the task. At the same time, some students in both groups, particularly in the Ineffective group, attempted to answer the question immediately after starting the task, consistent with their higher rates of ANSWER behaviors.

However, distinct structural divergences emerged. The Effective group (Figure 5a) generally displayed longer behavior sequences, characterized by sustained iterative cycles of designing and conducting experiments prior to committing to a final response. In contrast, the Ineffective group (Figure 5b) exhibited shorter sequences, and tended to answer earlier in the task.

Lastly, Table 3 summarizes the frequent subsequences (length ≤ 4) identified via cSPADE. Overall, the Effective group displayed a richer inquiry repertoire, exhibiting higher number of prominent patterns compared to the Ineffective group (22 vs. 8 patterns; see Table A3 for the full list).

The Effective group prioritized evidence collection from the outset. They predominantly initiated the task by designing and conducting experiments (e.g., <START, DESIGN, CONDUCT>, support = 0.58), whereas this initiation pattern was far less common in the Ineffective group (support = 0.27). Crucially, the Effective group engaged in sustained, iterative cycles of experimentation. The subsequence <DESIGN, CONDUCT> was nearly ubiquitous in the Effective group (support = 0.94) and frequently appeared in chained loops (e.g., <DESIGN, CONDUCT, DESIGN, CONDUCT>, support = 0.77), indicating patterns of continuous evidence accumulation before drawing conclusions.

In contrast, the Ineffective group exhibited distinct patterns of premature closure. They were more likely to attempt an answer immediately upon entering the task (e.g., <START, ANSWER>, support = 0.61 vs. 0.27 for Effective). Furthermore, their inquiry processes were often fragmented by rapid guessing behaviors. Unlike the Effective group who typically positioned answering at the end of long experimental chains, the Ineffective group frequently interspersed answer attempts before experimentation (e.g., <ANSWER, DESIGN, CONDUCT>, support = 0.51).

### 4.2. Micro-Level Inquiry Processes by Efficiency Within Effectiveness Groups (RQ2)

Descriptive statistics of micro-level task completion time and sequence length across the four performance subgroups are summarized in Table 4. Within the Effective group, the Effective–Efficient subgroup completed the task faster and with shorter sequences (time: *M* = 81.01, *SD* = 27.58; length: *M* = 11.92, *SD* = 4.40), than the Effective–Inefficient subgroup (time: *M* = 196.71, *SD* = 45.59; length: *M* = 20.50, *SD* = 8.14). Likewise, within the Ineffective group, the Ineffective–Efficient subgroup completed the task faster and with shorter sequences (time: *M* = 62.09, *SD* = 20.05; length: *M* = 7.86, *SD* = 3.08) than the Ineffective–Inefficient subgroup (time: *M* = 144.09, *SD* = 42.63; length: *M* = 9.55, *SD* = 3.40). These differences were significant for both time and sequence length in the Effective group (time: *t*(14.90) = −9.18, *p* < 0.001; length: *t*(14.51) = −3.84, *p* = 0.002) and in the Ineffective group (time: *t*(22.37) = −8.88, *p* < 0.001; length: *t*(26.24) = −2.19, *p* = 0.037).

Given the relatively small sample sizes of the inefficient profiles (Effective–Inefficient: *n* = 14; Ineffective–Inefficient: *n* = 22), the subsequent micro-level analyses are best interpreted as descriptive and exploratory.

**Figure 6:**
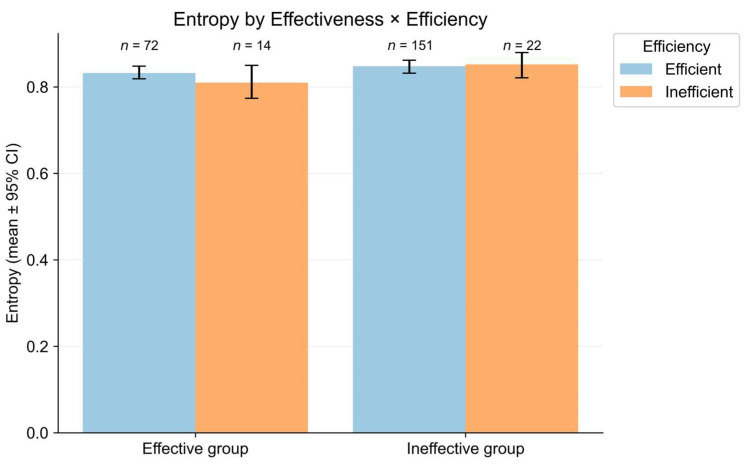
Mean behavioral entropy of micro-level inquiry processes across four performance subgroups. Error bars represent bootstrapped 95% confidence intervals of the mean. Numbers above bars indicate sample sizes for each performance subgroup.

Next, to quantify the diversity of micro-level behavior distributions, we examined behavioral entropy. As shown in Figure 6, bootstrapped mean entropy with 95% confidence intervals was remarkably consistent across the four performance subgroups, with means ranging from 0.81 to 0.85. Specifically, within the Effective group, the Effective–Efficient subgroup showed entropy comparable to the Effective–Inefficient subgroup (Effective–Efficient: *M* = 0.83, 95% CI [0.82, 0.85]; Effective–Inefficient: *M* = 0.81, 95% CI [0.78, 0.86]). Similarly, within the Ineffective group, the two subgroups exhibited similar mean entropy (Ineffective–Efficient: *M* = 0.85, 95% CI [0.83, 0.86]; Ineffective–Inefficient: *M* = 0.85, 95% CI [0.82, 0.88]).

A two-way ANOVA on the normalized entropy index confirmed this uniformity, revealing no statistically significant main effects of effectiveness (F(1, 255) = 3.05, *p* = 0.08, $\eta_{p}^{2}$ = 0.012), or efficiency (F(1, 255) = 0.14, *p* = 0.70, $\eta_{p}^{2}$ < 0.001), and no significant interaction (F(1, 255) = 0.73, *p* = 0.39, $\eta_{p}^{2}$ = 0.003). These results indicate that behavioral diversity alone was not discriminatory for performance in this task.

**Figure 7:**
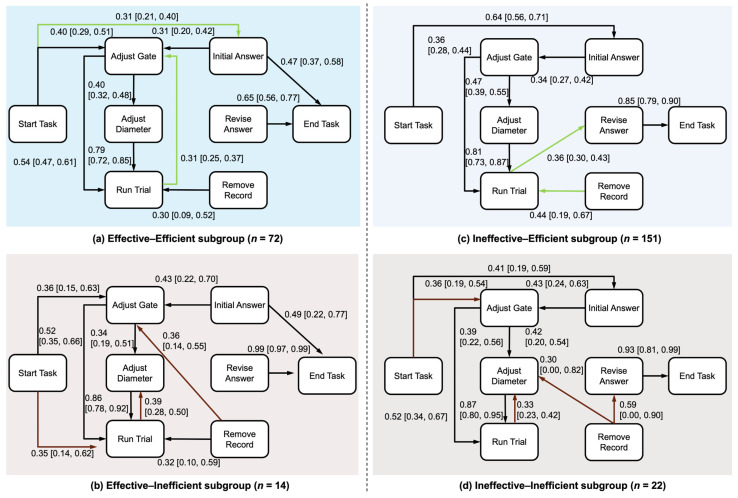
Process models of micro-level inquiry behaviors for the four performance subgroups: (**a**) Effective–Efficient, (**b**) Effective–Inefficient, (**c**) Ineffective–Efficient, and (**d**) Ineffective–Inefficient. Numbers on edges represent conditional transition probabilities (*Pr*). Only edges with *Pr* ≥ 0.30 are displayed for clarity. Within each effectiveness group, edges highlighted in green indicate transitions that are more prominent in the Efficient profile than in the corresponding Inefficient profile, whereas edges highlighted in red indicate transitions that are more prominent in the Inefficient profile.

**Figure 8:**
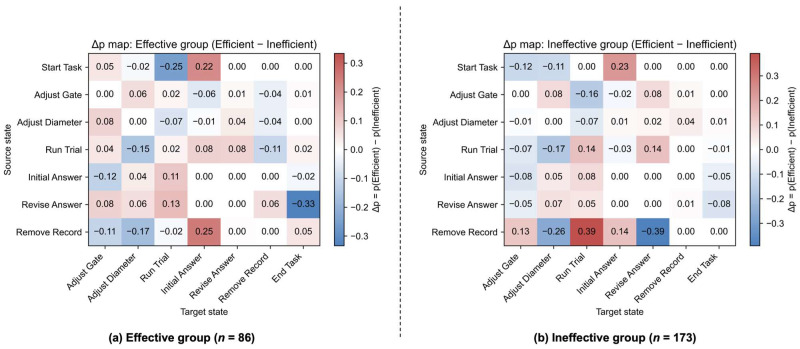
Delta probability maps (Efficient minus Inefficient) for (**a**) the Effective group and (**b**) the Ineffective group. Positive values indicate transitions that are more frequent in the Efficient profile, whereas negative values indicate transitions that are more frequent in the Inefficient profile.

Seeking further insight into the sequential structure of students’ micro-level behaviors, we applied process mining to estimate transition probabilities for each performance subgroup (Figure 7 and Figure 8). Across all four subgroups, the process models shared a common backbone reflecting the task’s experimental logic: Students typically transitioned from variable adjustments to running trials (Adjust Gate → Run Trial, *Prs* ≥ 0.36; Adjust Diameter → Run Trial, *Prs* ≥ 0.79), and eventually to ending the task after revising their answers (Revise Answer → End Task, *Prs* ≥ 0.65).

The central question for these exploratory micro-level analyses is how performance subgroups organized their behaviors around this backbone. A primary structural distinction emerged in the role of gate adjustments. Effective students integrated gate adjustments into iterative cycles (Effective–Efficient: Run Trial → Adjust Gate, *Pr* = 0.31, 95% CI [0.25, 0.37]; Effective–Inefficient: Remove Record → Adjust Gate, *Pr* = 0.36, 95% CI [0.14, 0.55]), whereas less effective students primarily transitioned into gate adjustments via one-way transitions (Ineffective–Efficient: Initial Answer → Adjust Gate, *Pr* = 0.34, 95% CI [0.27, 0.42]; Ineffective–Inefficient: Start Task → Adjust Gate, *Pr* = 0.36, 95% CI [0.19, 0.54]). Besides, less effective students also showed strong transitions from Start Task to Initial Answer (Ineffective–Efficient: *Pr* = 0.64, 95% CI [0.56, 0.71]; Ineffective–Inefficient: *Pr* = 0.41, 95% CI [0.19, 0.59]). These tentative patterns are broadly consistent with the task design, where the optimal solution requires coordinated adjustments of both gate and diameter settings.

Within the Effective group (Figure 7a,b), the two efficiency profiles differed in how they initiated and coordinated their behaviors towards the optimal condition. The Effective–Efficient subgroup predominantly initiated the task by proposing a tentative answer (Start Task → Initial Answer, *Pr* = 0.31, 95% CI [0.21, 0.40]), whereas the Effective–Inefficient subgroup was more likely to begin by running a trial with default settings (Start Task → Run Trial, *Pr* = 0.35, 95% CI [0.14, 0.62]), a pattern with low informative value.

The delta probability map for the Effective group (Figure 8a, Efficient minus Inefficient) is consistent with this contrast, with the full set of delta probabilities and 95% confidence intervals reported in Table A4. The Effective–Efficient subgroup was more likely to transition from Start Task to a tentative answer rather than directly running a default trial (Start Task → Initial Answer, Δ*p* = 0.22, 95% CI [0.03, 0.36]; Start Task → Run Trial, Δ*p* = −0.25, 95% CI [−0.51, −0.02]). They were also more likely to transition from initial answering to running trials (Initial Answer → Run Trial, Δ*p* = 0.11, 95% CI [0.04, 0.17]; Revise Answer → Run Trial, Δ*p* = 0.13, 95% CI [0.04, 0.22]). In contrast, the Effective–Inefficient subgroup more frequently routed transitions from running trials to adjusting diameter or deleting record (Run Trial → Adjust Diameter, Δ*p* = −0.15, 95% CI [−0.29, −0.02]; Run Trial → Remove Record, Δ*p* = −0.11, 95% CI [−0.19, −0.03]) and were more likely to finish the task immediately after revising their answer (Revise Answer → End Task, Δ*p* = −0.33, 95% CI [−0.43, −0.22]). Taken together, these patterns suggest that effective students ultimately reached the optimal condition, but the Effective–Efficient subgroup likely achieved this through focused experiments, whereas the Effective–Inefficient subgroup relied more heavily on local variable adjustments and record deletion, which may have contributed to their prolonged inquiry processes.

Within the Ineffective group (Figure 7c,d), the two efficiency profiles also differed in how they initiated the task and organized subsequent behaviors. While both sometimes answered immediately, the Ineffective–Efficient subgroup tended to transition directly to an initial answer (Start Task → Initial Answer, *Pr* = 0.64, 95% CI [0.56, 0.71]), whereas the Ineffective–Inefficient subgroup was more likely to begin by gate adjustments (Start Task → Adjust Gate, *Pr* = 0.36, 95% CI [0.19, 0.54]). This contrast in early moves was also reflected in transitions following trials and record deletions. After running a trial, the Ineffective–Efficient subgroup more often transitioned to revise their answer (Run Trial → Revise Answer, *Pr* = 0.36, 95% CI [0.30, 0.43]), whereas the Ineffective–Inefficient subgroup more often transitioned to adjusting diameter (Run Trial → Adjust Diameter, *Pr* = 0.33, 95% CI [0.23, 0.42]). When deleting records, the Ineffective–Efficient subgroup typically showed a transition to Run Trial (Remove Record → Run Trial, *Pr* = 0.44, 95% CI [0.19, 0.67]) whereas the Ineffective–Inefficient subgroup tentatively showed transitions both to diameter adjustments (Remove Record → Adjust Diameter, *Pr* = 0.30, 95% CI [0.00, 0.82]) and to answer revision (Remove Record → Revise Answer, *Pr* = 0.59, 95% CI [0.00, 0.90]).

The delta probability map for the Ineffective group (Figure 8b) also supports these group differences, with the full set of delta probabilities and 95% confidence intervals reported in Table A5. The Ineffective–Efficient subgroup was more likely to answer quickly by transitioning directly from Start Task to an initial answer (Start Task → Initial Answer, Δ*p* = 0.23, 95% CI [0.01, 0.45]), to run consecutive trials without changing variable setting (Run Trial → Run Trial, Δ*p* = 0.14, 95% CI [0.04, 0.23]), and to transition directly from deleting record to running trial (Remove Record → Run Trial, Δ*p* = 0.39, 95% CI [0.15, 0.62]). In contrast, the Ineffective–Inefficient subgroup was more likely to route transitions from running trials to adjusting diameter (Run Trial → Adjust Diameter, Δ*p* = −0.17, 95% CI [−0.27, −0.05]). Overall, both Ineffective–Efficient and Ineffective–Inefficient subgroups relied heavily on early answers and on trials that primarily adjusted diameter settings; critically, their subsequent trials, variable adjustments, and revisions lacked the systematic organization required to form the informative set of variable comparisons necessary for identifying the optimal condition.

## 5. Discussion

This study examined elementary students’ inquiry processes in a simulation-based inquiry task. By integrating macro-level analyses (e.g., sequential pattern mining) and micro-level analyses (e.g., process mining), we identified distinct behavioral patterns at both levels that were associated with students’ task performance in terms of effectiveness and efficiency.

### 5.1. Macro-Level Inquiry Patterns Across Effectiveness Groups

In response to RQ1, we examined macro-level behavioral frequencies, overall inquiry progression, and frequent subsequences to compare inquiry patterns between the Effective and Ineffective groups. Descriptive statistics and Poisson GLMs showed that the Effective group engaged more frequently in experiment design, execution, and data management than the Ineffective group. These findings indicate that effective students’ inquiry processes were more evidence-oriented, aligning with previous work that supports experimentation and reflection in interactive environments (Lin et al., 2023; Pedaste et al., 2015). Furthermore, these results corroborate prior work using aggregated process metrics, which has shown that high-performing students tend to engage more deeply in systematic exploration, whereas low-performing students exhibit limited or ineffective inquiry behaviors (Gobert et al., 2015; Teig et al., 2020). In contrast, the Ineffective group showed higher frequencies of answering behaviors, implying a tendency to respond prematurely without sufficient experimentation.

Sequence visualizations further highlighted marked differences in inquiry progression across effectiveness groups. While students in both groups anchored their inquiry around designing and conducting experiments, the Effective group generally exhibited longer behavior sequences characterized by frequent transitions between these phases. This pattern reflects iterative cycles where experimental designs were continuously refined based on empirical data. Such distinct iterative patterns align with prior studies emphasizing the reciprocal relationship between evidence collection and evaluation in scientific inquiry (Arnold et al., 2014; Emden & Sumfleth, 2016). In contrast, the Ineffective group tended to provide answers early in the task, indicating less deliberate exploration and potential difficulties in leveraging empirical evidence to support conclusions. Additionally, a subset of students in both effectiveness groups, though fewer among the Effective group, answered before engaging in experimentation, which may reflect an act-before-thinking style of interaction documented in simulation-based inquiry tasks (Teig, 2024). For effective students, however, this pattern could alternatively indicate that they drew on prior knowledge embedded in the task context rather than responding entirely at random, although this interpretation should be viewed with caution as we did not explicitly measure students’ prior content knowledge.

Sequential pattern mining substantiated these observations by uncovering frequent subsequences distinct to the Effective group, such as <DESIGN, CONDUCT, DESIGN> and <CONDUCT, DESIGN, CONDUCT>. These patterns point to a systematic alternation between planning and conducting experiments. In contrast, the Ineffective group showed frequent subsequences indicative of premature closure, such as <START, ANSWER> and <ANSWER, DESIGN, CONDUCT>, suggesting that their experimentation was often reactive to initial answers rather than proactively planned.

Overall, converging evidence from the macro-level analyses indicates that effective students demonstrated systematic inquiry processes characterized by iterative experimentation. Conversely, less effective students engaged in limited experimentation iterations and tended toward premature answering. These differences underscore the importance of iterative cycles of evidence collection and evaluation as a hallmark of successful inquiry (Emden & Sumfleth, 2016; Kranz et al., 2023; Metz, 2004).

### 5.2. Micro-Level Inquiry Patterns by Efficiency Within Effectiveness Groups

In response to RQ2, we analyzed micro-level behavioral entropy and transition probabilities to explore how efficiency further differentiated students’ inquiry processes within each effectiveness group. The intersection of effectiveness and efficiency dimensions resulted in four performance subgroups: Effective–Efficient (*n* = 72), Effective–Inefficient (*n* = 14), Ineffective–Efficient (*n* = 151), and Ineffective–Inefficient (*n* = 22). Notably, the sample sizes for the Effective–Inefficient and Ineffective–Inefficient subgroups were relatively limited. While this imbalance may constrain the statistical power of certain micro-level comparisons—particularly in process mining—the identified probabilistic transitions offer valuable insights into the dominant patterns within these specific profiles. Nevertheless, these findings should be interpreted with caution and warrant replication in larger samples and across diverse inquiry tasks to ensure generalizability.

Descriptive statistics for task completion time and sequence length indicate that, in this task, efficiency was associated with more streamlined inquiry. Within the Effective group, the Effective–Inefficient subgroup produced longer behavior sequences, reflecting relatively prolonged exploration that appeared to gradually converge toward the optimal condition. In contrast, while both efficiency profiles within the Ineffective group showed relatively short sequences, the less efficient students spent considerably more time on the task. Consistent with similar findings from prior research (Taub et al., 2018), the Effective–Efficient subgroup tended to minimize redundant behaviors and reduce time on task. Conversely, the Ineffective–Inefficient subgroup tended to engage in relatively ill-structured exploration, potentially signaling difficulties in evidence management.

Entropy analyses further revealed that students across the four performance subgroups exhibited a similarly broad repertoire of micro-level behaviors. The normalized entropy index ranged from 0.81 to 0.85, and a two-way ANOVA with effectiveness and efficiency as between-subjects factors yielded no statistically significant main effects or interaction. Therefore, behavioral diversity alone did not substantially differentiate inquiry processes in this task. This result underscores that inquiry performance may depend more on the behavioral coordination than on mere behavioral diversity.

Process mining showed that all four performance subgroups shared a common backbone, broadly aligning with the macro-level characterization of inquiry as iterative cycles of designing and conducting experiments. However, the specific ways in which students organized behavioral transitions around this backbone differed by effectiveness and efficiency. These results are in line with previous research in complex problem solving documenting substantial heterogeneity in behavioral patterns underlying both correct and incorrect solutions (Eichmann et al., 2020; Ulitzsch et al., 2022).

Within the Effective group, the Effective–Efficient subgroup showed organized transitions linking tentative answers to experimentation, where answer states cycled into trials and subsequently into variable adjustments. This pattern may reflect strategic, iterative adjustments across variables to locate the optimal condition. In contrast, the Effective–Inefficient subgroup appeared to rely heavily on default trials followed by diameter adjustments and record deletions, which could indicate a less coordinated approach toward the optimal solution. These patterns are consistent with the view that skilled scientific thinking requires sophisticated coordination of multiple variables (Kuhn et al., 2008).

Overall, micro-level analyses suggest that efficiency does not imply the adoption of distinct behaviors. Instead, effective and efficient students organized common behaviors into tighter, more purposeful cycles. We interpret this finding to mean that inquiry performance depends on the coordination of behavioral repertoires rather than the mere presence or absence of specific behaviors. These micro-level results complement the macro-level analyses, tentatively suggesting that productive inquiry requires not only engagement in evidence collection and evaluation but also the strategic coordination needed to orchestrate these inquiry phases coherently.

### 5.3. Implications

This study illustrates how process data from simulation-based inquiry tasks can be analyzed at multiple levels of granularity to derive interpretable evidence regarding students’ inquiry behaviors. Employing a multi-analytic approach, we integrate complementary techniques to extract both macro- and micro-level behavioral patterns and examine how these patterns relate to inquiry performance in terms of effectiveness and efficiency.

First, while prior research on scientific inquiry has predominantly emphasized effectiveness (i.e., task correctness), far fewer studies have examined efficiency, defined as the economical utilization of behavior and time resources (Taub et al., 2018; Wang et al., 2023). By jointly considering effectiveness and efficiency as complementary performance dimensions, the present study captures not only whether the processes lead to correct solutions, but also how economically students organize their behaviors within the processes.

Second, building on prior work on log-based inquiry assessment, this study shows the advantages of analyzing simulation-based process data within a multi-analytic macro–micro framework. While existing research has typically focused either on macro-level or micro-level sequences in isolation, recent reviews underscore the critical need to bridge these levels (e.g., Goldhammer et al., 2021; Lindner & Greiff, 2023). Our analyses integrate these perspectives by identifying macro-level inquiry patterns that distinguish systematic, iterative experimentation from premature closure and by complementing them with micro-level patterns that highlight the strategic coordination within evidence collection and evaluation. By synthesizing these two levels of granularity, we offer a comprehensive account of how structural coherence (macro-level) and strategic coordination (micro-level) jointly contribute to scientific inquiry performance.

Third, whereas most process-based inquiry studies have drawn on secondary or postsecondary samples (C. M. Chen & Wang, 2020; Teig, 2024), this study extends the scope of research to middle childhood. By investigating elementary students’ inquiry behaviors within a simulation-based environment, we demonstrate that distinct behavioral patterns linked to effectiveness and efficiency are discernible even at this early developmental stage. These findings provide empirical evidence for the early ontogeny of scientific inquiry skills (Metz, 2004; Y. Zheng et al., 2024).

Finally, from a practical standpoint, these findings characterize distinct performance profiles that can inform the design of process-based diagnostic assessments and targeted scaffolding. Specifically, our 2 × 2 performance dimensions crossing effectiveness with efficiency offers a robust basis for tailoring instruction to specific learner needs. For example, less effective yet efficient students tend to end exploration quickly and converge prematurely on incorrect solutions, suggesting a need for prompts that inhibit premature closure and encourage comprehensive evidence collection. Conversely, less effective and less efficient students, characterized by prolonged but ill-structured experimentation, require interventions focused on fostering systematic exploration strategies and logical hypothesis testing.

### 5.4. Limitations and Future Directions

This study has several limitations that suggest directions for future research. First, regarding methodology, we employed a multi-analytic approach including sequential analyses, entropy analysis, and process mining to examine behavioral patterns of scientific inquiry in relation to task performance. Although these techniques yielded rich insights, they rely on specific assumptions about temporal dependence and data sparsity and only capture selected aspects of the underlying processes. Future work could integrate additional analytic perspectives, such as psychometric models for simulation-based assessments that combine product and process data (e.g., Bayesian networks) and complex dynamical systems approaches that use network metrics to characterize trajectories over time (De Klerk et al., 2015; S. Li et al., 2025). The use of machine learning approaches to process data may also help identify nonlinear patterns and improve predictive validity, provided that resulting models are carefully validated and interpreted (Zhang et al., 2025).

Second, as technological advances broaden the modalities of process data that can be collected, future research may benefit from incorporating additional data sources to complement log file analyses. Multimodal data, such as self-report questionnaires (Ober et al., 2021), think-aloud protocols (Pohl et al., 2016; Vieira et al., 2018), and eye-tracking techniques (Chiou et al., 2022; Jian et al., 2024), can provide convergent evidence on students’ cognitive and metacognitive processes during inquiry and help validate inferences drawn from log-based process data alone (Molenaar et al., 2023).

Third, the behavioral patterns identified here should be generalized with caution. Although we combined multiple analytic techniques, all results were derived from process data from a single simulation-based inquiry task in one content domain. Future work should examine whether similar behavioral transitions emerge across diverse inquiry environments and task designs and should investigate the consistency of students’ behavioral patterns across tasks, domains, and populations (Kang & Liu, 2022; Bhaw et al., 2023; He et al., 2021).

Finally, we operationalized inquiry performance using task effectiveness (correctness) and efficiency (task completion time), which captured only part of the multifaceted nature of inquiry skills. Behaviors that are not directly reflected in these outcomes, such as strategic planning, monitoring, and self-regulation, merit further investigation. Future work could employ process-based performance rubrics that incorporate behavioral and temporal metrics (R. S. Baker et al., 2016; Gobert et al., 2013) to assess inquiry performance beyond absolute correctness and time on task.

Considering this information, and factoring in its exploratory nature, this study provides an initial foundation for understanding how students’ inquiry patterns relate to task effectiveness and efficiency. Future research that leverages additional analytic methods, multimodal data sources, and cross-context designs will be essential for validating and extending these findings.

## Figures and Tables

**Table 1:** Coding scheme for behaviors in the Hydroelectric Power Plant inquiry task.

Cognitive Component	Macro-Level Behavior	Micro-Level Behavior	Description
Evidence Collection	DESIGN	Adjust Gate	The student adjusts the gate position (Low/Medium/High) in the Experimentation Panel.
		Adjust Diameter	The student adjusts the inlet diameter (40 cm/80 cm/120 cm) in the Experimentation Panel.
	CONDUCT	Run Trial	The student clicks the “Run” button to conduct an experimental trial; the system automatically records the resulting Gate × Diameter condition in the Data Panel.
Evidence Evaluation	ANSWER	Initial Answer	The student provides a first response for this question in the Question Panel.
		Revise Answer	The student later returns to the Question Panel and modifies a previous response for this question.
	MANAGE	Remove Record	The student deletes a recorded row of data in the Data Panel.
Task Control	START	Start Task	The student enters the task (the first logged behavior event for this task).
	END	End Task	The student clicks the “End” button to end the task and submits final answers.

**Table 2:** Descriptive statistics and Poisson GLMs of macro-level inquiry behaviors.

Behavior	Effectiveness Group					
	Effective (*n* = 86)		Ineffective (*n* = 173)			
	*M*	*SD*	*M*	*SD*	IRR (95% CI)	*p*
START	1.00	0.00	1.00	0.00	—	—
DESIGN	3.74	2.63	1.52	1.33	1.53 [1.38, 1.69]	<0.001
CONDUCT	4.20	2.58	2.01	1.60	1.30 [1.19, 1.42]	<0.001
ANSWER	1.62	0.74	1.82	0.61	0.56 [0.48, 0.65]	<0.001
MANAGE	0.49	0.89	0.16	0.51	1.88 [1.10, 3.20]	0.020
END	1.00	0.00	1.00	0.00	—	—

*Note.* IRR = incidence rate ratio. Abbreviations: START = Start the task; DESIGN = Design experiments; CONDUCT = Conduct experiments; ANSWER = Answer the question; MANAGE = Manage data; END = End the task.

**Table 3:** Frequent subsequences of macro-level inquiry processes across effectiveness groups.

Subsequences	Support (95%CI)		Student Count		*p*
	Effective	Ineffective	Effective	Ineffective	
Frequent subsequences primarily for the Effective group					
<START, DESIGN>	0.59 [0.49, 0.69]	0.34 [0.27, 0.41]	51	59	<0.001
<START, DESIGN, CONDUCT>	0.58 [0.48, 0.68]	0.27 [0.21, 0.34]	50	47	<0.001
<START, DESIGN, CONDUCT, DESIGN>	0.55 [0.44, 0.65]	0.11 [0.08, 0.17]	47	20	<0.001
<CONDUCT, DESIGN>	0.83 [0.73, 0.89]	0.29 [0.23, 0.36]	71	51	<0.001
<CONDUCT, DESIGN, CONDUCT>	0.79 [0.69, 0.86]	0.27 [0.21, 0.34]	68	47	<0.001
<CONDUCT, DESIGN, CONDUCT, ANSWER>	0.57 [0.46, 0.67]	0.18 [0.13, 0.25]	49	32	<0.001
<CONDUCT, DESIGN, CONDUCT, DESIGN>	0.51 [0.41, 0.61]	0.13 [0.08, 0.18]	44	22	<0.001
<DESIGN, CONDUCT>	0.94 [0.87, 0.97]	0.78 [0.71, 0.83]	81	135	0.001
<DESIGN, CONDUCT, DESIGN>	0.80 [0.71, 0.87]	0.25 [0.19, 0.32]	69	44	<0.001
<DESIGN, CONDUCT, DESIGN, CONDUCT>	0.77 [0.67, 0.84]	0.23 [0.17, 0.30]	66	40	<0.001
Frequent subsequences primarily for the Ineffective group					
<START, ANSWER>	0.27 [0.19, 0.37]	0.61 [0.54, 0.68]	23	107	<0.001
<START, ANSWER, DESIGN>	0.23 [0.16, 0.33]	0.43 [0.36, 0.51]	20	75	0.002
<START, ANSWER, DESIGN, CONDUCT>	0.21 [0.14, 0.31]	0.38 [0.31, 0.45]	18	66	0.007
<ANSWER, DESIGN, CONDUCT>	0.35 [0.26, 0.45]	0.51 [0.43, 0.58]	30	88	0.021
<ANSWER, DESIGN, CONDUCT, ANSWER>	0.13 [0.07, 0.21]	0.34 [0.28, 0.42]	11	60	<0.001

*Note.* Abbreviations: START = Start the task; DESIGN = Design experiments; CONDUCT = Conduct experiments; ANSWER = Answer the question; MANAGE = Manage data; END = End the task. Sample sizes were *n* = 86 for the Effective group and *n* = 173 for the Ineffective group. Frequent subsequences are defined as those with support ≥ 0.30 and *p* < 0.05 in at least one group. Only subsequences with length ≤ 4 are presented. Within each panel, subsequences are ordered by their starting behavior and length.

**Table 4:** Descriptive statistics of micro-level task completion time and sequence length across four performance subgroups.

Effectiveness Group	Efficiency Profile	Completion Time		Sequence Length	
		*M*	*SD*	*M*	*SD*
Effective (*n* = 86)	Efficient (*n* = 72)	81.01	27.58	11.92	4.40
	Inefficient (*n* = 14)	196.71	45.59	20.50	8.14
Ineffective (*n* = 173)	Efficient (*n* = 151)	62.09	20.05	7.86	3.08
	Inefficient (*n* = 22)	144.09	42.63	9.55	3.40

*Note.* Task completion time is measured in seconds. Sequence length refers to the total number of micro-level behaviors recorded per student.

## Data Availability

The dataset analyzed in this study was collected in 2019 by the Collaborative Innovation Center of Assessment for Basic Education Quality at Beijing Normal University as part of a large-scale educational project on students’ scientific inquiry skills. In accordance with institutional regulations on educational data security, test confidentiality, and participant privacy, the original log files and full test materials cannot be made publicly available. De-identified data sufficient to reproduce the main analyses are available from the corresponding author upon reasonable request. Anonymized synthetic process logs together with all analysis scripts, allowing reproduction of the full analysis workflow, are openly available in Open Science Framework (OSF) at https://osf.io/my35c/overview (13 December 2025).

## Abbreviations

The following abbreviations are used in this manuscript:

GLM	Generalized Linear Model
GMM	Gaussian Mixture Model
IRR	Incidence Rate Ratio

## Appendix B

**Table A1:** Fit indices (BIC and AIC) for GMMs with 1-4 components by effectiveness group.

Effectiveness Group	*K*	BIC	AIC
Effective (*n* = 86)	1	934.55	929.64
	2	917.57	905.29
	3	929.61	909.97
	4	937.26	910.26
Ineffective (*n* = 173)	1	1743.74	1737.44
	2	1693.91	1678.14
	3	1699.21	1673.98
	4	1709.10	1674.42

**Table A2:** Descriptive statistics of $P_{DR}$ across alternative efficiency groupings.

Effectiveness Group	Grouping Method	Efficiency Group	*n* (%)	*M*	*SD*
Effective (*n* = 86)	Median split	Efficient	27 (31%)	0.14	0.10
		Inefficient	59 (69%)	0.16	0.09
	Tertiles	Most efficient (T1)	12 (14%)	0.10	0.11
		Middle efficient (T2)	37 (43%)	0.14	0.09
		Least efficient (T3)	37 (43%)	0.18	0.08
Ineffective (*n* = 173)	Median split	Efficient	107 (62%)	0.10	0.09
		Inefficient	66 (38%)	0.16	0.09
	Tertiles	Most efficient (T1)	53 (31%)	0.08	0.09
		Middle efficient (T2)	91 (53%)	0.13	0.09
		Least efficient (T3)	29 (17%)	0.18	0.08

*Note.* $P_{DR}$ is the proportion of Adjust Diameter → Run Trial transitions. Efficiency groups were defined based on task completion time within each effectiveness group using median-split and tertile groupings.

## Appendix C

**Table A3:** Full set of frequent subsequences of macro-level inquiry processes across effectiveness groups.

Subsequences	Support (95%CI)		Student Count		*p*
	Effective	Ineffective	Effective	Ineffective	
Frequent subsequences primarily for the Effective group					
<START, DESIGN>	0.59 [0.49, 0.69]	0.34 [0.27, 0.41]	51	59	<0.001
<START, DESIGN, CONDUCT>	0.58 [0.48, 0.68]	0.27 [0.21, 0.34]	50	47	<0.001
<START, DESIGN, CONDUCT, DESIGN>	0.55 [0.44, 0.65]	0.11 [0.08, 0.17]	47	20	<0.001
<START, DESIGN, CONDUCT, DESIGN, CONDUCT>	0.51 [0.41, 0.61]	0.10 [0.07, 0.16]	44	18	<0.001
<START, DESIGN, CONDUCT, DESIGN, CONDUCT, DESIGN>	0.30 [0.22, 0.41]	0.05 [0.02, 0.09]	26	8	<0.001
<CONDUCT, DESIGN>	0.83 [0.73, 0.89]	0.29 [0.23, 0.36]	71	51	<0.001
<CONDUCT, DESIGN, CONDUCT>	0.79 [0.69, 0.86]	0.27 [0.21, 0.34]	68	47	<0.001
<CONDUCT, DESIGN, CONDUCT, ANSWER>	0.57 [0.46, 0.67]	0.18 [0.13, 0.25]	49	32	<0.001
<CONDUCT, DESIGN, CONDUCT, DESIGN>	0.51 [0.41, 0.61]	0.13 [0.08, 0.18]	44	22	<0.001
<CONDUCT, DESIGN, CONDUCT, ANSWER, END>	0.44 [0.34, 0.55]	0.17 [0.12, 0.23]	38	29	<0.001
<CONDUCT, DESIGN, CONDUCT, DESIGN, CONDUCT>	0.50 [0.40, 0.60]	0.12 [0.08, 0.18]	43	21	<0.001
<CONDUCT, DESIGN, CONDUCT, DESIGN, CONDUCT, ANSWER>	0.31 [0.23, 0.42]	0.08 [0.05, 0.13]	27	14	<0.001
<CONDUCT, DESIGN, CONDUCT, DESIGN, CONDUCT, DESIGN>	0.31 [0.23, 0.42]	0.03 [0.02, 0.07]	27	6	<0.001
<CONDUCT, DESIGN, CONDUCT, DESIGN, CONDUCT, DESIGN, CONDUCT>	0.31 [0.23, 0.42]	0.03 [0.01, 0.07]	27	5	<0.001
<DESIGN, CONDUCT>	0.94 [0.87, 0.97]	0.78 [0.71, 0.83]	81	135	0.001
<DESIGN, CONDUCT, DESIGN>	0.80 [0.71, 0.87]	0.25 [0.19, 0.32]	69	44	<0.001
<DESIGN, CONDUCT, DESIGN, CONDUCT>	0.77 [0.67, 0.84]	0.23 [0.17, 0.30]	66	40	<0.001
<DESIGN, CONDUCT, DESIGN, CONDUCT, ANSWER>	0.55 [0.44, 0.65]	0.16 [0.11, 0.22]	47	28	<0.001
<DESIGN, CONDUCT, DESIGN, CONDUCT, DESIGN>	0.45 [0.35, 0.56]	0.11 [0.08, 0.17]	39	20	<0.001
<DESIGN, CONDUCT, DESIGN, CONDUCT, ANSWER, END>	0.42 [0.32, 0.52]	0.14 [0.10, 0.20]	36	25	<0.001
<DESIGN, CONDUCT, DESIGN, CONDUCT, DESIGN, CONDUCT>	0.44 [0.34, 0.55]	0.11 [0.07, 0.16]	38	19	<0.001
Frequent subsequences primarily for the Ineffective group					
<START, ANSWER>	0.27 [0.19, 0.37]	0.61 [0.54, 0.68]	23	107	<0.001
<START, ANSWER, DESIGN>	0.23 [0.16, 0.33]	0.43 [0.36, 0.51]	20	75	0.002
<START, ANSWER, DESIGN, CONDUCT>	0.21 [0.14, 0.31]	0.38 [0.31, 0.45]	18	66	0.007
<ANSWER, DESIGN, CONDUCT>	0.35 [0.26, 0.45]	0.51 [0.43, 0.58]	30	88	0.021
<ANSWER, DESIGN, CONDUCT, ANSWER>	0.13 [0.07, 0.21]	0.34 [0.28, 0.42]	11	60	<0.001
<ANSWER, DESIGN, CONDUCT, ANSWER, END>	0.13 [0.07, 0.21]	0.34 [0.27, 0.41]	11	59	<0.001

*Note.* Abbreviations: START = Start the task; DESIGN = Design experiments; CONDUCT = Conduct experiments; ANSWER = Answer the question; MANAGE = Manage data; END = End the task. Sample sizes were *n* = 86 for the Effective group and *n* = 173 for the Ineffective group. Frequent subsequences are defined as those with support ≥ 0.30 and *p* < 0.05 in at least one group. Within each panel, subsequences are ordered by their starting behavior and length.

## Appendix D

**Table A4:** Delta probabilities (Efficient minus Inefficient) with 95% confidence intervals for the Effective group.

	Source	Adjust Gate	Adjust Diameter	Run Trial	Initial Answer	Revise Answer	Remove Record	End Task
Target								
Start Task		0.05 [−0.23, 0.32]	−0.02 [−0.25, 0.20]	−0.25 [−0.51, −0.02]	0.22 [0.03, 0.36]	—	—	—
Adjust Gate		—	0.06 [−0.14, 0.23]	0.02 [−0.14, 0.20]	−0.06 [−0.13, 0.01]	0.01 [−0.00, 0.03]	−0.04 [−0.11, 0.03]	0.01 [−0.00, 0.02]
Adjust Diameter		0.08 [−0.01, 0.17]	—	−0.07 [−0.17, 0.02]	−0.01 [−0.05, 0.02]	0.04 [0.01, 0.07]	−0.04 [−0.10, 0.02]	—
Run Trial		0.04 [−0.06, 0.16]	−0.15 [−0.29, −0.02]	0.02 [−0.08, 0.12]	0.08 [0.03, 0.13]	0.08 [0.01, 0.15]	−0.11 [−0.19, −0.03]	0.02 [0.01, 0.04]
Initial Answer		−0.12 [−0.41, 0.14]	0.04 [−0.13, 0.16]	0.11 [0.04, 0.17]	—	—	—	−0.02 [−0.29, 0.27]
Revise Answer		0.08 [0.02, 0.16]	0.06 [−0.00, 0.13]	0.13 [0.04, 0.22]	—	—	0.06 [−0.00, 0.14]	−0.33 [−0.43, −0.22]
Remove Record		−0.11 [−0.38, 0.17]	−0.17 [−0.37, 0.02]	−0.02 [−0.35, 0.33]	0.25 [0.02, 0.49]	0.00 [−0.10, 0.14]	—	0.05 [−0.00, 0.15]

**Table A5:** Delta probabilities (Efficient minus Inefficient) with 95% confidence intervals for the Ineffective group.

	Source	Adjust Gate	Adjust Diameter	Run Trial	Initial Answer	Revise Answer	Remove Record	End Task
Target								
Start Task		−0.12 [−0.33, 0.07]	−0.11 [−0.28, 0.04]	0.00 [−0.11, 0.08]	0.23 [0.01, 0.45]	—	—	—
Adjust Gate		—	0.08 [−0.11, 0.27]	−0.16 [−0.34, 0.03]	−0.02 [−0.15, 0.07]	0.08 [0.04, 0.13]	0.01 [−0.00, 0.04]	—
Adjust Diameter		−0.01 [−0.10, 0.09]	—	−0.07 [−0.17, 0.04]	0.01 [−0.00, 0.03]	0.02 [−0.00, 0.04]	0.04 [0.01, 0.09]	0.01 [−0.00, 0.02]
Run Trial		−0.07 [−0.14, 0.00]	−0.17 [−0.27, −0.05]	0.14 [0.04, 0.23]	−0.03 [−0.13, 0.06]	0.14 [0.02, 0.26]	0.00 [−0.07, 0.06]	−0.01 [−0.07, 0.03]
Initial Answer		−0.08 [−0.31, 0.14]	0.05 [−0.11, 0.19]	0.08 [−0.08, 0.22]	—	—	—	−0.05 [−0.27, 0.15]
Revise Answer		−0.05 [−0.16, 0.03]	0.07 [0.03, 0.11]	0.05 [0.02, 0.09]	—	—	0.01 [−0.00, 0.02]	−0.08 [−0.18, 0.04]
Remove Record		0.13 [−0.03, 0.34]	−0.26 [−0.77, 0.08]	0.39 [0.15, 0.62]	0.14 [0.01, 0.30]	−0.39 [−0.77, 0.23]	—	—
